# Sintering Distortion in Multi-Composition-Layered Zirconia Disks for Dental Prostheses: An Experimental Analysis

**DOI:** 10.3390/ma18184234

**Published:** 2025-09-09

**Authors:** Mizuho Hirano, Syuntaro Nomoto, Hideshi Sekine

**Affiliations:** Department of Fixed Prosthodontics, Tokyo Dental College, 2-9-18 Kanda-Misakicho, Chiyoda-ku, Tokyo 101-0061, Japan; nomotos@tdc.ac.jp (S.N.); sekine@tdc.ac.jp (H.S.)

**Keywords:** zirconia, distortion, multi-layered, sintering, milling-area

## Abstract

Zirconia is a high-strength ceramic and has increasing importance, particularly in the field of aesthetic dentistry for crown restorations. Multi-composition-layered-type (MCL) zirconia is attracting attention as a new material that integrates high light transmittance with mechanical strength. However, there are no reports on the deformation induced by sintering in MCL zirconia. Therefore, we aimed to investigate the sintering distortion of MCL zirconia. An experimental fixed dental prosthesis (FDP) was designed based on a 4-unit monolithic zirconia FDP. A MCL with no color gradation and an MCL with color gradation were selected. Particularly, three milling areas—the top end of the disk (area I) (n = 7), vertical center (area II) (n = 7), and bottom end of the disk (area III) (n = 7)—were investigated. Moreover, sintering distortions generated by experimental FDPs were measured. Sintering distortion was detected in all areas. The direction of distortion varied by area—positive in area I, negative in area II, and approximately zero in area III—with a significant difference between areas I and II (*p* = 0.001). The largest absolute distortion was observed in c-MCL-A (area I); the corresponding marginal gaps were ~89.4 μm (second molar) and ~56.9 μm (first premolar), both below the clinical threshold of 120 μm.

## 1. Introduction

Zirconia has attracted attention as a dental material that can replace metal ions [1,2]. It is used in various applications, such as denture bases [3] and orthodontic brackets [4], owing to its comparable mechanical strength to metals and aesthetic appeal. Several clinical studies have been conducted on employing zirconia as a dental crown restoration material that can be used on a daily basis [5,6,7]. However, to perform aesthetic dental crown restoration, it is necessary to mimic the shade and translucency of natural teeth. Therefore, the aesthetic appearance of dental crowns has been enhanced by veneering feldspar-based ceramic materials or glass onto zirconia [8].

The usefulness of monolithic zirconia restorations has attracted considerable attention in recent years [8]. Such restorations are used in cases where tooth preparation poses challenges, particularly in cases involving teeth wherein securing the amount of cutting or managing a high bite force is problematic. Single-composition-layered zirconia, a disk made of pure zirconia that displays gradation from the lighter shade of the incisal edge to the darker shade of the cusp, is one of the materials that plays a role in this process. This composition contains Fe, which gives zirconia an ivory color [9]. Shade gradation is caused by adjusting the Fe concentration in stages and presenting layers. Many previous studies have used single-composition-layered zirconia [10,11]. Our previous study [12] suggested that the layered structure of single-composition-layered zirconia affected the sintering distortion of 4-unit fixed dental prostheses (FDPs). The addition of different concentrations of Fe to zirconia, which has a high shrinkage rate during post-sintering, causes a shrinkage imbalance.

Additionally, the low light transmittance of conventional zirconia poses another challenge [13]. In cases where a high level of aesthetic quality is required, reproducing the translucency of the occlusal surface and incisal edge is necessary. To meet this need, highly transparent zirconia with a high yttria content has been developed. Zirconia with 5% yttria (5Y-PSZ), also known as the cubic zirconia crystal (which is optically isotropic), accounts for 50% of the entire crystal. Consequently, 5Y-PSZ has 20–25% better light transmittance than the zirconia with 3% yttria (3Y-TZP) [14]. However, the concern of decreased mechanical strength owing to the addition of yttria has been raised. According to a study that examined the zirconia flexural strength, 3Y-TZP had a flexural strength of 1194 ± 111 MPa, whereas 5Y-PSZ had a flexural strength of 688 ± 159 MPa [15]. Furthermore, because 5Y-PSZ is highly translucent, the effect of the shade of the abutment tooth on the crown color was also noted [16]. When the surface of a decayed or discolored tooth is covered, masking the shade of the abutment tooth becomes necessary [17].

**Figure 1:**
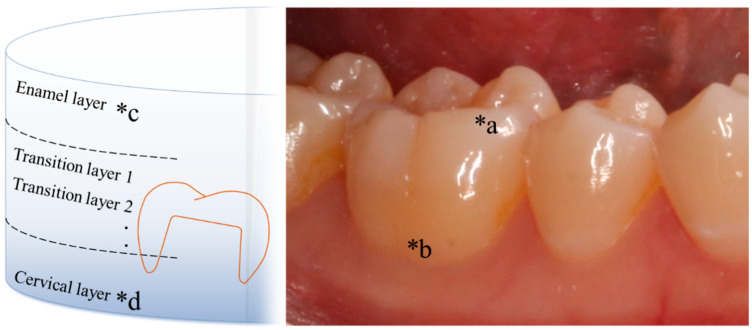
Color gradation of multi-composition-layered type (MCL) disks and natural teeth. (*a) The position close to the occlusal surface of the teeth is relatively lighter in color and more translucent. (*b) The closer the tooth is to the cervical layer, the darker the relative color and the more mechanical strength required. (*c) The enamel layer of the disk has a relatively high yttria content and is highly translucent. (*d) The cervical layer of the disk has relatively low yttria content and high mechanical strength. Adapted from Figure 3 in [12] (J. Mech. Behav. Biomed. Mater. 2022, 135, 105078).

A newly developed product is a multi-composition-layered-type (MCL) zirconia [18]. In MCL zirconia, highly translucent zirconia is used on the cutting edge and occlusal surfaces, whereas low-translucent zirconia is used on the cervical side (Figure 1). This makes it possible to create dental crown prostheses that are more aesthetic than single-composition-layered prostheses. Generally, 4–5% yttria-containing zirconia is used on the incisal side, whereas 3–4% yttria-containing zirconia is used on the cervical side. Furthermore, MCL disks with additional shade gradation were developed by adding Fe to the layers. MCL zirconia is being developed as an aesthetic crown material, with new products being released consecutively by various manufacturers [14].

While several studies have been conducted on the deformation caused by sintering in single-composition-layered zirconia, no reports exist on the sintering distortion of MCL zirconia. This knowledge may assist dental professionals in optimizing the positioning of restorations within zirconia disks during CAD/CAM fabrication. Furthermore, a better understanding of position-dependent sintering behavior could support the development of more predictable restorative protocols and improve clinical outcomes in zirconia-based prosthodontics.

To examine this further, the present study aimed to investigate the effects of the layered structure and vertical processing area on the sintering distortion of MCL zirconia.

Our null hypotheses were as follows:

1. MCL zirconia disks do not produce distortion during the post-sintering process.
2. The difference in the vertical processing area of the MCL zirconia disk does not cause a difference in sintering distortion.

## 2. Materials and Methods

### 2.1. Design of the Test FDPs

**Figure 2:**
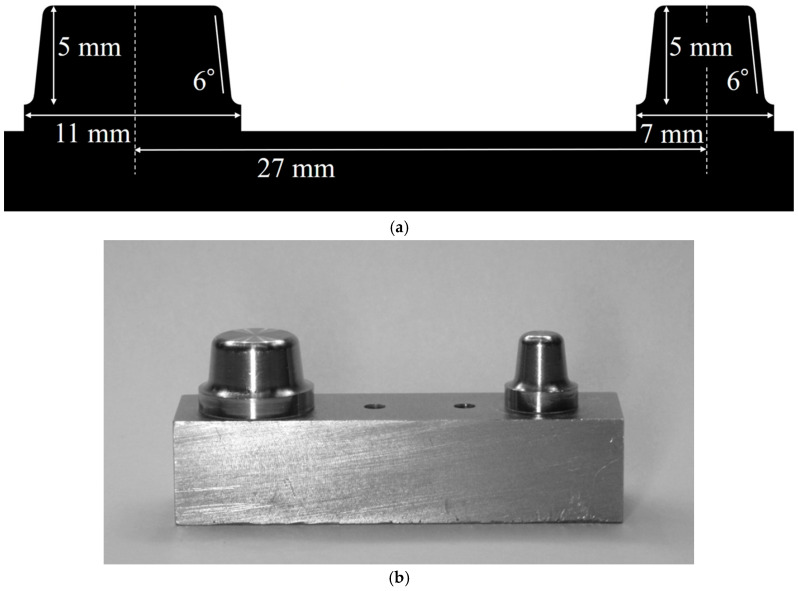
Experimental abutment tooth model. (**a**) Schematic design of the experimental abutment tooth model. (**b**) Appearance of the experimental abutment tooth model. Adapted from Figure 4 in [12] (J. Mech. Behav. Biomed. Mater. 2022, 135, 105078).

A stainless steel model was prepared with the second bicuspid and first molar missing and abutment teeth formed for all-ceramic crowns on the first bicuspid and second molar (Figure 2).

**Figure 3:**
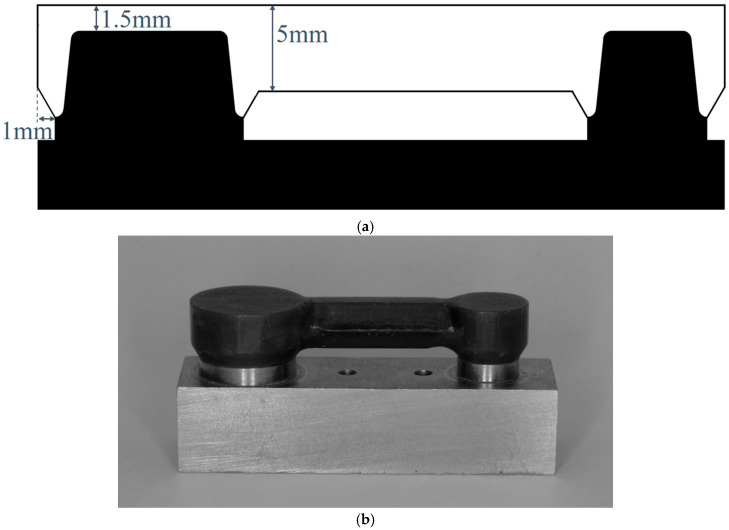
Experimental fixed dental prostheses (FDPs), assuming monolithic zirconia FDPs, were waxed up. (**a**) Schematic design of experimental FDPs. (**b**) Wax pattern of experimental FDPs. Adapted from Figure 5 in [12] (J. Mech. Behav. Biomed. Mater. 2022, 135, 105078).

The widths of the abutment teeth were 7 and 11 mm for the first and second molars, respectively, and the height was 5 mm for both abutment teeth. The abutment teeth have deep chamfers with a radius of curvature of 1 mm. Waxing of the experimental FDPs was performed using the model illustrated in Figure 3.

The experimental FDPs were simplified versions of a 4-unit monolithic zirconia bridge. The lateral surfaces of the abutment devices had a cylindrical shape 1 mm outside the margin. The emergence profile was raised 60° above the margin and connected in a cylindrical form on the side. The occlusal surface thickness was 1.5 mm. The retainers were connected using a hexagonal pillar with a cross-sectional area of 22.5 mm^2^, which was used as the connector and pontic.

The outer shape of the stainless steel model and the wax pattern were scanned using a laboratory scanner (S-WAVE Scanner D2000, 3SHAPE, Copenhagen, Denmark). Each value was converted to STL data. Using a dental CAD software (Dental Manager ver.2.102.1.0, 3Shape, Copenhagen, Denmark), the data for each were superimposed to create experimental FDP data.

### 2.2. Milling and Scanning of Experimental FDPs

#### 2.2.1. Milling of Experimental FDPs

**Figure 4:**
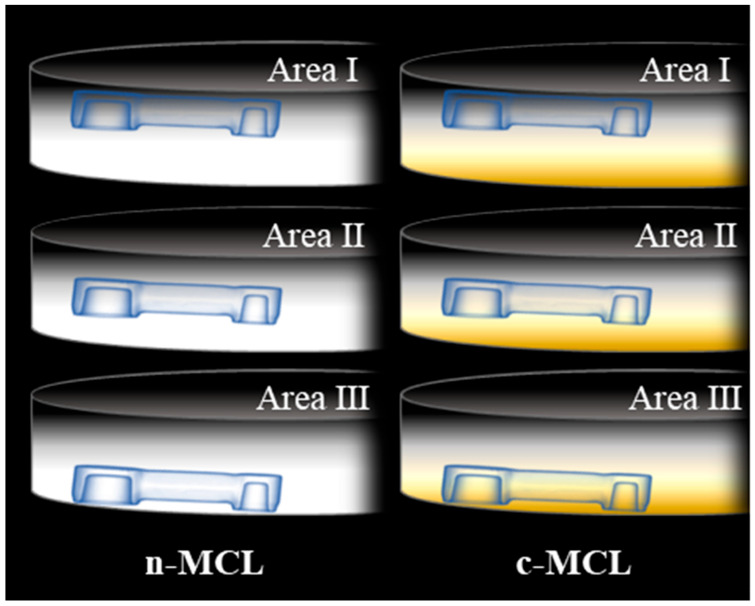
Vertical milling area of the experimental fixed dental prosthesis (FDP) in the zirconia disk. Area I is positioned above the disk, Area II is positioned vertically in the center of the disk, and Area III is positioned below the disk. The distances from the disk surface to Areas I and III are each 0.1 mm.

The zirconia disks used are listed in Table 1. We selected a MCL with no color gradation (n-MCL; the ‘n’ indicates no color) and an MCL with color gradation (c-MCL; the ‘c’ indicates color) from each manufacturer. For c-MCL, we selected the VITA Classical Shade A3. The areas for milling were as follows: area I, the enamel-colored side of the disk (0.1 mm inside the surface of the disk); area II, the vertical center of the disk; and area III, the cervical-colored side of the disk (0.1 mm inside the surface of the disk) (Figure 4).

To minimize variability, all specimens for areas I–III within each product (n-MCL and c-MCL) were milled from the same production lot per manufacturer. The manufacturer-specified sintering magnification factor for each disc is reported in Table 1. Milling strategies, toolpaths and the furnace program were identical across areas.

#### 2.2.2. Scanning of the Milled Experimental FDPs

The pre-sintered experimental FDPs were scanned using a laboratory scanner (S-WAVE Scanner D2000). These FDPs were used as the control group (c) (n = 7). The use of a laboratory scanner for pre-/post-sintering STL acquisition is consistent with current accuracy studies in crown fit analysis [19].

### 2.3. Post-Sintering and Scanning of Experimental FDPs

#### 2.3.1. Post-Sintering

**Figure 5:**
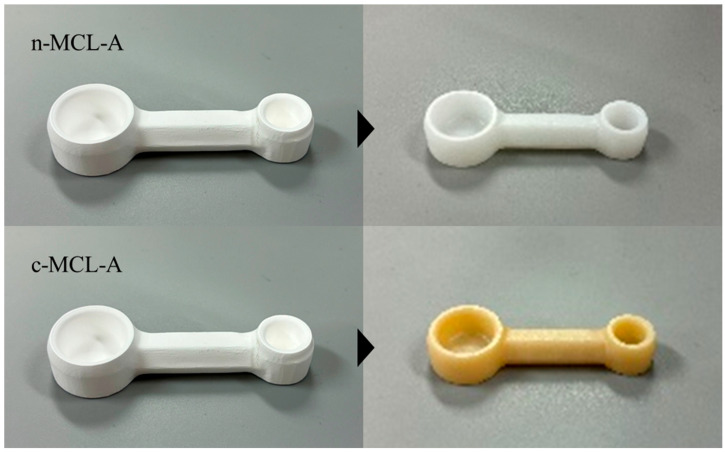
Pre- and post-sintering experimental FDPs. The experimental FDPs were ultrasonically cleaned after post-sintering.

The experimental FDPs were post-sintered in a zirconia furnace (Esthemat Sinta II, Matsufu, Kyoto, Japan), following the manufacturer’s instructions (Table 2). The post-sintering FDPs were ultrasonically cleaned in water for 5 min. Figure 5 shows the experimental FDPs before and after sintering.

#### 2.3.2. Scanning of Post-Sintered Experimental FDPs

The experimental FDPs were scanned one by one using a dental laboratory scanner (S-Wave Scanner D2000) and converted to STL data. This constituted the experimental group (e) (n = 7).

### 2.4. Digital Measurement of Experimental FDPs Before and After Post-Sintering

In addition to the method for measuring the sintering distortion of FDPs devised in our previous study [12], we created a color map using the best-fit method and observed the distortion.

**Figure 6:**
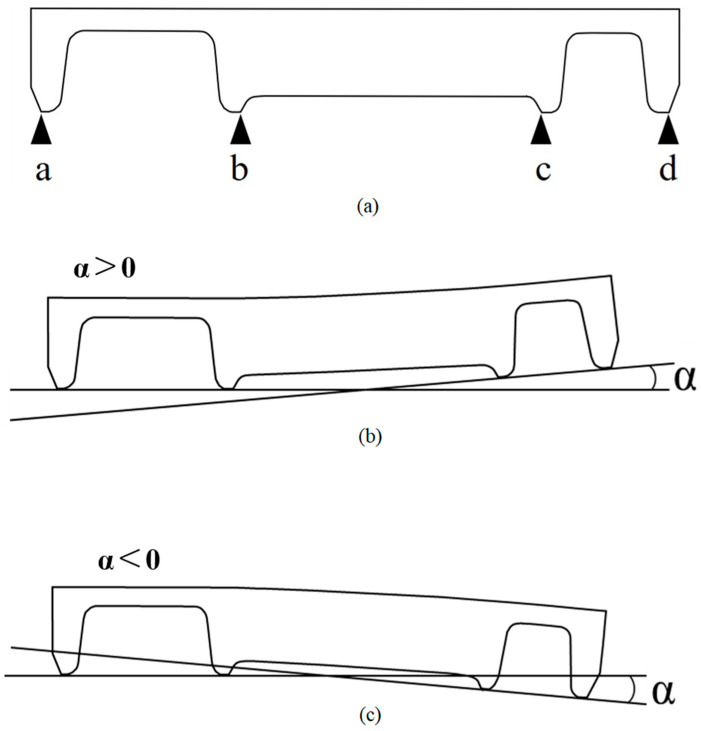
Margin measurement points and angle α measurement points. The angles of the pre- and post-sintered experimental fixed dental prostheses (FDPs) are α(c) and α(e), respectively, and the difference between them, α(e–c), is calculated. This is determined to be the sintering distortion caused by the post-sintering. (**a**) Margin measurement points. The measurement points are on the mesiodistal margins of both retainers in the buccolingual central cross-section of the experimental FDPs. Two points are connected by a line to each retainer. (**b**) Measurement points when angle α > 0, that is, when the two lines intersect on the root side. (**c**) Measurement points when angle α < 0, that is, when the two lines intersect on the crown side. Adapted from Figure 10 in [12] (J. Mech. Behav. Biomed. Mater. 2022, 135, 105078).

The STL data of the control and experimental groups were imported into the CAD software (Fusion360^TM^, AutoDesk Inc., San Rafael, CA, USA). The buccal-lingual midsagittal cross-section of the experimental FDPs was observed at a magnification of 910×, and the margin measurement points (a–d) contained in the cross-sectional image were marked (Figure 6a). Here, a, b, c, and d were connected to draw two straight lines. The external angle α formed by the two lines was measured using the software. The cases where the intersection point was on the root side of the retainer margin were recorded as α > 0 (Figure 6b), and the cases where the intersection point was on the crown side were recorded as α < 0 (Figure 6c). The measured values were recorded for each experimental FDP.

The difference between the control group angle α(c) and the corresponding experimental group angle α(e) was calculated. This was attributed to the sintering distortion caused by post-sintering.

Scan data of the post-sintered test FDPs were enlarged according to the disk’s magnification factor (Fusion360^TM^, AutoDesk Inc., San Rafael, CA, USA). Enlarged scan data and the corresponding scan data of the pre-sintered test FDPs were superimposed using the best-fit method (Cloud Compare V2, Daniel Girardeau-Montaut). Post-sintering STL data were registered to pre-sintering data using a best-fit (iterative closest point) superimposition, and color-coded 3D deviation maps were generated to visualize area-dependent distortion—an approach widely applied in dental metrology to assess the accuracy of CAD/CAM prostheses [20,21,22].

### 2.5. Statistical Analysis

First, the angles α(c) of pre-sintering and α(e) of post-sintering in each group were compared using the Wilcoxon signed-rank test (n = 7). Second, the sintering distortion α(e–c) of areas I, II, and III in each disk were compared using the Kruskal–Wallis test and Dunn–Bonferroni multiple comparison test (n = 7). Finally, the sintering distortion α(e–c) of n-MCL and c-MCL obtained from the same manufacturer was compared using the Wald–Wolfowitz run test, with the three areas of each disk as a group (n = 21). The significance level was set at 5% using the statistical software SPSS (version 27, IBM Corp., Armonk, NY, USA).

## 3. Results

Table 3 and Table 4 show the comparison of the angle α(c) of the pre-sintering experimental FDPs and the angle α(e) of the post-sintering experimental FDPs in each processing area of the disk. The vertical axis shows the angle α(c and e), and the horizontal axis shows each processing area. A significant difference between the angle α(c) and the angle α(e) was observed in all areas.

**Figure 7:**
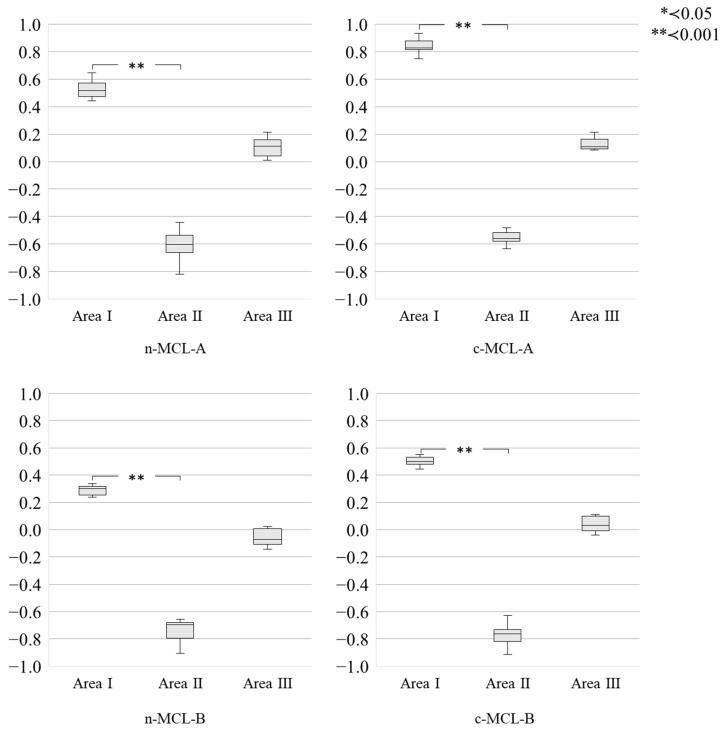
Comparison of sintering distortion α(e–c) for each area. The horizontal axes represent areas, and the vertical axes represent the sintering distortion α(e–c). The sintering distortion α(e–c) in each area is compared using the Kruskal–Wallis and Dunn–Bonferroni tests.

The sintering distortion α(c–e) for each area was compared for each disk (Figure 7). For n-MCL and c-MCL, positive sintering distortion was observed in area I. By contrast, negative sintering distortion was observed in area II. A significant difference was observed between areas I and II (n-MCL-A, *p* = 0.001; n-MCL-B, *p* = 0.001; c-MCL-A, *p* = 0.001; and c-MCL-B, *p* = 0.001). In area III, the values were close to zero, and the sintering distortion was smaller than that in areas I and II. Across all experimental FDPs, the maximum sintering distortion α(e–c) recorded was 0.932° (in area I of c-MCL-A).

**Figure 8:**
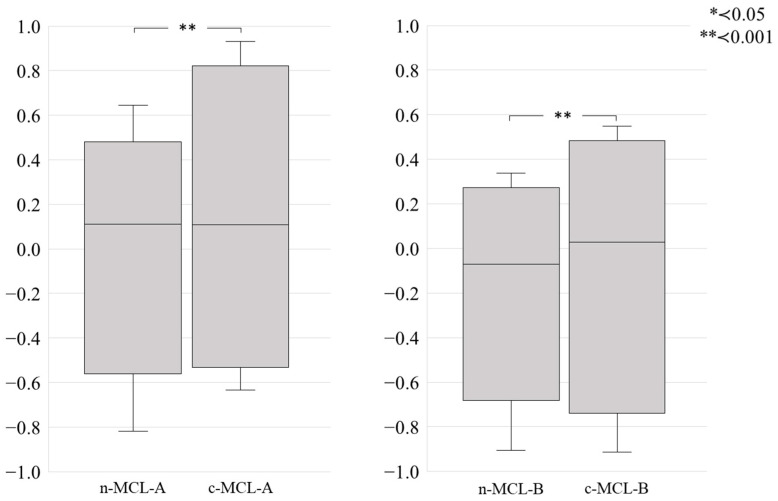
Comparison of sintering distortion α(e–c) on a colorless disk (n-MCL) and a disk with a color gradient (c-MCL) disk. The sintering distortion α(e–c) in both disk types is compared using the Wald–Wolfowitz runs tests.

Furthermore, when comparing n-MCLs and c-MCLs, the variation between the areas was greater for c-MCLs (A, *p* = 0.001; B, *p* = 0.001) (Figure 8).

**Figure 9:**
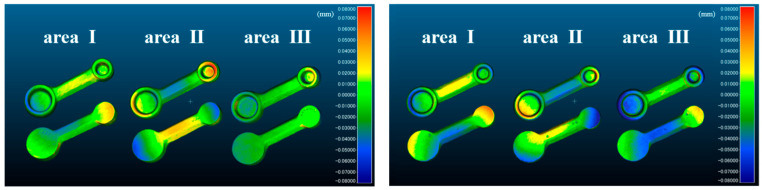
Distortion of FDPs post sintering is shown in a color map compared to that of FDPs pre sintering. Red indicates convex sintering distortion, and blue indicates concave sintering distortion.

Figure 9 shows a color map indicating the distortion caused by sintering. Both n-MCL and c-MCL showed the same trend. In regions 1 and 3, distortion occurred in the occlusal direction. In region 2, distortion occurred in the cervical direction.

## 4. Discussion

### 4.1. Selection of Zirconia Disks

This study selected MCL zirconia disks from commercially available zirconia disks utilized in routine clinical practice. These disks can be used as 4-unit FDPs in the molar region. In our previous study, we noted that single-composition-layered-type zirconia undergoes sintering distortion. Therefore, the same product from the same manufacturer was used for zirconia disks in this study. Furthermore, the thickness of the disks was selected to be 18 mm, which is an intermediate thickness among several available types.

According to manufacturer documentation and a recent peer-reviewed review, the KATANA Zirconia YML disc is constructed as a composition-gradient multilayer comprising 5Y-PSZ, 4Y-PSZ, and 3Y-TZP from the enamel to the cervical side. SHOFU Disk ZR Lucent Supra is likewise classified as a 3Y/4Y/5Y composition-gradient zirconia [23]. The exact layer-by-layer mol% Y_2_O_3_ values and Fe_2_O_3_ concentrations are not publicly disclosed by the manufacturers.

### 4.2. Rationale for the Experimental FDP Design

We deliberately adopted the same stainless steel model and 4-unit posterior FDP geometry as in our previous work to ensure continuity and to enable direct comparison of sintering-induced deformation across material systems and positioning strategies [12]. The design choices were made to isolate the effect of the multilayer composition and disk position from confounding geometric factors that are known to influence densification and shape change during sintering.

Specifically, the axial surfaces of the retainers were modelled as cylinders offset 1 mm from the finish line, the occlusal thickness was kept constant at 1.5 mm, and the retainers were joined by a prismatic, hexagonal connector–pontic element with a cross-sectional area of 22.5 mm^2^. This simplified, low-curvature morphology reduced local constraint and curvature-driven gradients, thereby minimizing geometry-dependent shrinkage differentials and warpage. In line with the general sintering theory and prior observations, complex contours and abrupt section changes tend to amplify non-uniform shrinkage, whereas prismatic elements better reflect the intrinsic material/position effects we sought to measure [24].

A 4-unit span was selected because it represents a clinically relevant posterior bridge length in which sintering distortion is measurable without introducing the additional variability associated with longer spans or intermediate abutments. Using a standardized, repetition-friendly design across disk areas I–III allowed us to attribute between-area differences primarily to the multilayer architecture (e.g., Y_2_O_3_ gradient and, where applicable, Fe-based shading layers) and vertical positioning within the disk, rather than to design heterogeneity.

### 4.3. Measurement

The sintering distortion of the experimental FDPs was evaluated by connecting the marginal areas of the abutment teeth with a line and measuring the angle formed by the two lines. This method uses the rule that ‘corresponding angles are equal in similar figures’. However, many studies that mention the distortion of FDP external shape have observed the fitness of the abutment teeth. Methods such as the replica method [25] and micro-CT scanning [25,26] are currently used. These methods are suitable for applying FDPs to abutment teeth in clinical practice; they can be considered appropriate for situations in clinical practice where FDPs are cemented to the abutment teeth.

### 4.4. Sintering Distortion

Zirconia undergoes significant volume shrinkage during sintering [23]. This shrinkage is caused by a decrease in the volume and porosity among zirconia-powder particles, with an increase in temperature [27]. However, this shrinkage is not always uniform. Therefore, predicting the shrinkage characteristics of zirconia is essential for manufacturing defect-free sintered bodies [28,29].

In our previous study [12], we measured the FDP sintering distortion in single-composition-type and single-composition-layered zirconia disks. In the former, we observed distortion owing to the shape of the FDPs. In the latter, we observed that the metal oxide layers affected the sintering distortion, whose magnitude differed in each area.

In the MCL examined in this study, sintering distortion also occurred (Table 3 and Table 4). Therefore, our null hypothesis (1)—that MCL zirconia disks do not produce sintering distortion during the post-sintering process—was rejected. In addition to the distortion caused by the shape of the FDPs identified in the single composition type [12], the gradient of the Y_2_O_3_ concentration was found to be related to the distortion. When Y_2_O_3_ is added to zirconia, the grain size of the zirconia powder increases, which causes differences in the densification process through sintering [30]. In general, the 3Y-TZP particles are finer than the 5Y-TZP particles and therefore have larger specific surface areas. Previous studies have reported that the higher the specific surface area, the higher the surface energy and the more rapid the sintering [31]. The final relative density achieved by sintering also differs depending on the concentration of Y_2_O_3_ added [32]. That is, an increase in the concentration of Y_2_O_3_ added to zirconia affects the shrinkage rate and speed. This indicates that the MCL zirconia contains zirconia with different shrinkage behaviors layered on top of each other [33].

Furthermore, alongside sintering distortion in each area, we observed differences in size and direction (Figure 7). Therefore, our null hypothesis (2)—that there is no difference in sintering distortion in the vertical processing area of the MCL zirconia disk—was rejected.

The experimental FDPs used in this study showed a large positive distortion in area I and a negative distortion in area II. This result contradicts our expectations. According to the manufacturer, the zirconia disks used in this study had a finely layered structure at the center. Thus, most of the FDPs in area I were machined from the 5Y layer, and only a small part near the margin contained a layered structure. However, the FDPs in area II contained all the layered structures in the disk, indicating that the difference in Y_2_O_3_ concentration was the greatest. Assuming that the difference in Y_2_O_3_ concentration causes sintering distortion, we predicted that the sintering distortion would be close to zero in area I and far from zero in area II. However, the results differed from our predictions. As the composition of the disk was not known in detail, it was not possible to say with certainty what caused the observed results. However, one of the causes may have been the difference in the shrinkage rate mentioned above or the ‘difference in the temperature at which shrinkage begins’. As the Y_2_O_3_ concentration in zirconia increases, the temperature at which shrinkage begins shifts to higher temperatures. According to a report by Chek Hai Lim et al. [34], when 3% Y_2_O_3_ is added, shrinkage begins at a lower temperature than when 4% or 5% Y_2_O_3_, and the peak shrinkage temperatures are 1025 °C for 3%, 1050 °C for 4%, and 1070 °C for 5% Y_2_O_3_. As most dental zirconia is processed from semi-sintered bodies that have been sintered at approximately 1000–1100 °C [35,36], the degree of sintering of the pre-sintered disks is expected to vary depending on Y_2_O_3_ concentrations. This result is consistent with the findings of the study by Harada et al. [37], which reported that a lower density of the pre-sintered body correlates with the increased shrinkage of the material following final sintering. That is, the semi-sintered MCL disks possibly contain layers with different degrees of sintering, presumably resulting in each layer displaying different shrinkage amounts during the post-sintering process. These factors are believed to interact in complex ways, thereby causing differences in the sintering distortion of each area.

Furthermore, compared with n-MCL, c-MCL displayed greater variation among areas (Figure 8). c-MCL exhibits both a transparency gradient and a color gradient, alongside a layered structure characterized by varying concentrations of Fe_2_O_3_, in addition to Y_2_O_3_. We believe that this factor leads to a greater variation among areas.

Based on the results of this study, we believe that areas II and III should be used to maximize the characteristics of the MCL and minimize sintering distortion. To achieve this, it may be necessary to choose a disk with a slightly lighter shade than desired and to place it closer to the cervix. However, this is only an assumption, as this study did not compare disks with different shades. Therefore, further research is required in this area. From a practical perspective, our results can inform dental technicians’ positioning strategy in CAD/CAM systems to reduce chairside adjustments and remakes due to marginal discrepancies. Clinicians should also consider mechanical implications across current zirconia generations, because flexural strength varies among materials (including multilayer systems) [38].

In addition to compositional gradients, differences in the pre-sintered state—such as relative density and pore structure—are known to modulate densification kinetics and final linear shrinkage. The absence of lot-specific green-state data may therefore partly account for the between-area variation observed here, consistent with prior observations [37].

### 4.5. Margin Gap

The application of zirconia disks with translucency and shade gradation enables aesthetic restoration using monolithic zirconia; therefore, it is widely used in clinical practice. However, in this study, a difference in the degree of sintering distortion due to the milling area was observed in the MCL disks, suggesting that translucency, shade adjustment, and sintering distortion must be considered when generating prosthetic devices.

A clinically acceptable marginal gap of 120 μm has been reported [39]. To relate the measured angular distortion (α) to linear marginal discrepancy, we assumed that the rotation was equally shared by the two retainers (θ = α/2) and approximated each abutment margin by a circle of diameter *D*. Under the small-angle approximation (α in radians), Δ ≈ *D*·(α/2). For the present model (second molar *D* = 11.0 mm; first premolar *D* = 7.0 mm) and α = 0.932° (0.0163 rad), the estimated marginal gaps were 89.4 μm and 56.9 μm, respectively—both below the commonly cited clinical threshold [39].

These calculations were specific to the 4-unit FDP geometry tested; longer spans or pier-abutment designs may increase marginal discrepancies [40]. Some manufacturers of MCL zirconia also specify indications/limitations; adherence to these constraints is advisable. Collectively, our findings suggest that the disc position is a clinically relevant factor for marginal adaptation and long-term restoration success. With advances in digital dentistry, incorporating material-positioning strategies may help standardize outcomes and reduce chairside adjustments.

### 4.6. Clinical Implications for Daily Restorative Practice

The present study was experimental and did not reproduce the full complexity of intraoral environment. Nevertheless, the observed position-dependent distortion supports several pragmatic steps for daily practice:

(i) When using multi-composition-layered (MCL) zirconia, posterior short-span FDPs should be preferentially positioned in Areas II–III of the disk to minimize distortion (Figure 7);
(ii) If cervical positioning compromises shade selection, one shade lighter should be considered before milling;
(iii) Uniform wall thickness should be maintained and abrupt section changes near connectors should be avoided to reduce geometry-driven warpage. These simple measures translate the experimental findings into routine CAD/CAM workflows and may reduce chairside adjustments and remakes while preserving the esthetic advantages of MCL zirconia.

Although the present study focused on position-dependent sintering distortion and did not assess biological outcomes, zirconia is generally considered highly biocompatible, supporting favorable soft-tissue responses when appropriately finished and polished [41]. Recent reviews [42] indicate comparable or improved gingival fibroblast behavior on zirconia compared with titanium and that biofilm adhesion is influenced by surface chemistry and roughness rather than the bulk ceramic itself. These points suggest that minimizing distortion-related marginal discrepancies remains clinically relevant not only for fit but also for plaque control and peri-restorative tissue health.

### 4.7. Limitations

This study has some limitations. When FDPs are placed on abutment teeth, the distortion in one area may be evaluated as a lack of fitness in another; it is not possible to evaluate the sintering distortion of the FDP itself. In particular, the horizontal contraction of zirconia crowns is greater than the vertical contraction [43], and the distortion that occurs in the margins and axial surfaces may manifest as a lack of fitness on the occlusal surface. Therefore, this study did not measure the fitness of the abutment teeth but rather observed the distortion of the experimental FDPs themselves. In the future, it will be necessary to measure the fitness of abutment teeth in a way that is more aligned with clinical practice, as this will facilitate the discussion and interpretation of the study results. Further studies incorporating different framework designs, veneering procedures, or intraoral scanning outcomes could provide a broader understanding of how sintering-induced distortion affects clinical outcomes.

The pre-sintered state density was not directly measured, and the manufacturers did not disclose this parameter. Because pre-sintered density and pore architecture can influence densification and distortion, this remains a limitation. We minimized confounding by milling n-MCL and c-MCL specimens from the same production lots, using identical CAD/CAM strategies, and sintering all specimens in one furnace under the same cycle.

These directions may broaden our understanding of clinical impact. At the material level, however, the study had a specific limitation: layer-resolved Y_2_O_3_ or Fe_2_O_3_ data for the lots tested were unavailable. This constraint prevented a direct test of the hypothesis that local composition gradients drive position-dependent distortion. To address this, future work will apply µ-Raman, XRD and EDS mapping to the same material batches to quantify the composition–distortion linkage.

## 5. Conclusions

In this study, MCL zirconia disks exhibited post-sintering distortion, and the direction and magnitude of the distortion differed in each area. However, the sintering distortion was limited, and in the experimental setting of this study, it did not exceed the range of clinical tolerance. These findings underscore the need to consider not only the material properties of MCL zirconia but also spatial positioning during CAD/CAM planning. Furthermore, the demonstrated positional effect on sintering distortion may have implications for reducing chairside adjustments, improving restoration longevity, and enhancing patient outcomes. Future research incorporating clinical fit evaluations and varying prosthetic designs could further support the optimization of zirconia-based restorative workflows.

## Figures and Tables

**Table 1:** Materials used.

Code	Product Name	Shade	Yttria Concentration (mol%)	Bending Strength (MPa)	Translucency (%)	Manufacturer	Lot No.	Sintering Magnification (Manufacturer-Specified)
n-MCL-A	ZR lucent Supra	Plain	3–5	1034–1454	37–44	Shofu (A)	DBU189G25H	1.231
c-MCL-A	ZR lucent Supra	A 3					DBU189H06A	1.231
n-MCL-B	Katana (YML)	NW	4–5	750–1100	45–49	Kraray Noritake (B)	EHGID	1.225
c-MCL-B	Katana (YML)	A 3					EGZJQ	1.225

Among mixed-composition-layered-type (MCL) disks, a colorless disk (n-MCL) and a disk with a color gradient (c-MCL) were used.

**Table 2:** Sintering program.

Code	Manufacturer	Sintering Conditions				
		Heating Rate	→	Plateau Temperature and Time	→	Cooling Rate
n-MCL-A	Shofu (A)	5 °C/min	→	1450 °C 120 min	→	−10 °C/min
c-MCL-A						
n-MCL-B	Kuraray Noritake (B)	10 °C/min	→	1500 °C 120 min	→	−10 °C/min
c-MCL-B						

Each experimental fixed dental prosthesis (FDP) was sintered according to the program recommended by the manufacturer.

**Table 3:** Comparison of pre-sintering and post-sintering angle α for each area on the n-MCL-type disk. *: Indicates a statistically significant difference.

		Angle α		Wilcoxon Signed-Rank Test
		Pre-Sintering α(c)	Post-Sintering α(e)	
A	Area I	0.001222884	0.562673630	*
	Area II	0.010474830	−0.601709081	*
	Area III	0.009570891	0.112128207	*
B	Area I	0.008267323	0.260082590	*
	Area II	0.005885941	−0.732170211	*
	Area III	0.007005436	−0.049290299	*

**Table 4:** Comparison of pre-sintering and post-sintering angle α for each area on the c-MCL-type disk. *: Indicates a statistically significant difference.

		Angle α		Wilcoxon Signed-Rank Test
		Pre-Sintering α(c)	Post-Sintering α(e)	
A	Area I	0.010930750	0.847735490	*
	Area II	0.012567790	−0.543305760	*
	Area III	0.016376560	0.142615717	*
B	Area I	0.001698136	0.501140251	*
	Area II	0.004838907	−0.766374026	*
	Area III	0.004772353	0.035230390	*

## Data Availability

The original contributions presented in this study are included in the article. Further inquiries can be directed to the corresponding author.

## Abbreviations

The following abbreviations are used in this manuscript:

FDP	fixed dental prostheses
MCL	multi-composition-layered type

## References

[1] Soleimani F., Jalali H., Mostafavi A.S., Zeighami S., Memarian M. (2020). Retention and clinical performance of zirconia crowns: A comprehensive review. Int. J. Dent..

[2] Aldhuwayhi S. (2025). Zirconia in dental implantology: A review of the literature with recent updates. Bioengineering.

[3] Muluk D.S., Grover D.I. (2023). Aesthetics of claps in removable partial denture—A literature review. Int. J. Adv. Dent. Sci. Technol..

[4] Babaee Hemmati Y., Neshandar Asli H., Falahchai M., Safary S. (2022). Effect of different surface treatments and orthodontic bracket type on shear bond strength of high-translucent zirconia: An in vitro study. Int. J. Dent..

[5] Alghauli M., Alqutaibi A.Y., Wille S., Kern M. (2024). 3D-printed versus conventionally milled zirconia for dental clinical applications: Trueness, precision, accuracy, biological and esthetic aspects. J. Dent..

[6] Abdelhafez A., Dhar V. (2025). Comparative clinical performance of stainless steel, zirconia, and Bioflx crowns in primary molars: A randomized controlled trial. BMC Oral Health.

[7] D’Souza N.L., Jutlah E.M., Deshpande R.A., Somogyi-Ganss E. (2025). Comparison of clinical outcomes between single metal-ceramic and zirconia crowns. J. Prosthet. Dent..

[8] Kontonasaki E., Giasimakopoulos P., Rigos A.E. (2020). Strength and aging resistance of monolithic zirconia: An update to current knowledge. Jpn. Dent. Sci. Rev..

[9] Mikeli A., Walter M.H., Rau S.A., Raedel M., Raedel M. (2022). Three-year clinical performance of posterior monolithic zirconia single crowns. J. Prosthet. Dent..

[10] Cho M.H., Seol H.J. (2022). Optical properties, microstructure, and phase fraction of multi-layered monolithic zirconia with and without yttria-gradient. Materials.

[11] Suzuki S., Katsuta Y., Ueda K., Watanabe F. (2020). Marginal and internal fit of three-unit zirconia fixed dental prostheses: Effects of prosthesis design, cement space, and zirconia type. J. Prosthodont. Res..

[12] Hirano M., Nomoto S., Sato T., Yotsuya M., Hisanaga R., Sekine H. (2022). Sintering distortion of monolithic zirconia in 4-unit fixed partial denture: Effect of layered structure and vertical milling area. J. Mech. Behav. Biomed. Mater..

[13] Sulaiman T.A., Abdulmajeed A.A., Donovan T.E., Ritter A.V., Lassila L.V., Vallittu P.K., Närhi T.O. (2015). Degree of conversion of dual-polymerizing cements light polymerized through monolithic zirconia of different thicknesses and types. J. Prosthet. Dent..

[14] Ban S. (2021). Classification and properties of dental zirconia as implant fixtures and superstructures. Materials.

[15] Kwon S.J., Lawson N.C., McLaren E.E., Nejat A.H., Burgess J.O. (2018). Comparison of the mechanical properties of translucent zirconia and lithium disilicate. J. Prosthet. Dent..

[16] Elkhodary N., Aboubakr K. (2021). Assessment of final shade and translucency of two monolithic zirconia versus veneered zirconia on a dark substrate. An Invitro study. Egypt. Dent. J..

[17] Cho Y.E., Lim Y.J., Han J.S., Yeo I.L., Yoon H.I. (2020). Effect of yttria content on the translucency and masking ability of yttria-stabilized tetragonal zirconia polycrystal. Materials.

[18] Badr Z., Culp L., Duqum I., Lim C.H., Zhang Y., A. Sulaiman T. (2022). Survivability and fracture resistance of monolithic and multi-yttria-layered zirconia crowns as a function of yttria content: A mastication simulation study. J. Esthet. Restor. Dent..

[19] Farag E.A.A., Rizk A., Ashraf R., Emad Eldin F. (2024). Effect of the scanner type on the marginal gap and internal fit of two monolithic CAD/CAM esthetic crown materials: An in vitro study. Dent. Med. Probl..

[20] Limones A., Molinero-Mourelle P., Çakmak G., Abou-Ayash S., Delgado S., Martínez Vázquez de Parga J.A., Celemín A. (2024). Impact of the superimposition methods and the designated comparison area on accuracy analyses in dentate models. J. Dent..

[21] Zhang T., Yang B., Ge R., Zhang C., Zhang H., Wang Y. (2024). Effect of a novel ‘scan body’ on the in vitro scanning accuracy of full-arch implant impressions. Int. Dent. J..

[22] Nulty A. (2022). A comparison of trueness and precision of 12 3D printers used in dentistry. BDJ Open.

[23] Cesar P.F., Miranda R.B.P., Santos K.F., Scherrer S.S., Zhang Y. (2024). Recent advances in dental zirconia: 15 years of material and processing evolution. Dent. Mater..

[24] Olevsky E.A. (1998). Theory of sintering: From discrete to continuum. Mater. Sci. Eng. R Rep..

[25] de Kok P., Liao P., Chien E.C., Morgano S. (2025). A meta-analysis of the accuracy of different measuring techniques to evaluate the marginal and internal gap of a fixed dental prosthesis: The American Academy of Fixed Prosthodontics, Research in Fixed Prosthodontics Committee. J. Prosthet. Dent..

[26] Daou E.E., Salameh P. (2024). Does the choice of the measuring technique affect the comparison of fit between zirconia and cobalt-chromium prostheses?. J. Indian Prosthodont. Soc..

[27] Lee C.Y., Lee S., Ha J.H., Lee J., Song I.H., Moon K.S. (2021). Effect of the sintering temperature on the compressive strengths of reticulated porous Zirconia. Appl. Sci..

[28] Coldea A., Meinen J., Hoffmann M., Elsayed A., Stawarczyk B. (2025). Shrinkage behavior of strength-gradient multilayered zirconia materials. Materials.

[29] Manière C., Chan S., Lee G., McKittrick J., Olevsky E.A. (2018). Sintering dilatometry based grain growth assessment. Results Phys..

[30] Alves M.F.R.P., Ribeiro S., Suzuki P.A., Strecker K., dos Santosc C. (2021). Effect of Fe_2_O_3_ addition and sintering temperature on mechanical properties and translucence of zirconia dental ceramics with different Y_2_O_3_ content. Mater. Res..

[31] Matsui K., Tanaka K., Yamakawa T., Uehara M., Enomoto N., Hojo J. (2007). Sintering kinetics at isothermal shrinkage: II, effect of Y_2_O_3_ concentration on the initial sintering stage of fine zirconia powder. J. Am. Ceram. Soc..

[32] Alves M.F.R.P., Dos Santos C., Elias C.N., Amarante J.E.V., Ribeiro S. (2023). Comparison between different fracture toughness techniques in zirconia dental ceramics. J. Biomed. Mater. Res. B Appl. Biomater..

[33] Kongkiatkamon S., Rokaya D., Kengtanyakich S., Peampring C. (2023). Current classification of zirconia in dentistry: An updated review. PeerJ.

[34] Lim C.H., Vardhaman S., Reddy N., Zhang Y. (2022). Composition, processing, and properties of biphasic zirconia bioceramics: Relationship to competing strength and optical properties. Ceram. Int..

[35] Qian H., Cui C., Su T., Zhang F., Sun J. (2016). Exploring the optimal pre-sintering temperature on compressive strength and anti-fatigue property of graded zirconia-based glass/zirconia structure. Dent. Mater. J..

[36] Kastyl J., Stastny P., Chlup Z., Song L., Scasnovic E., Trunec M. (2022). Gelcast zirconia ceramics for dental applications combining high strength and high translucency. J. Am. Ceram. Soc..

[37] Harada A., Shishido S., Barkarmo S., Inagaki R., Kanno T., Örtengren U., Egusa H., Nakamura K. (2020). Mechanical and microstructural properties of ultra-translucent dental zirconia ceramic stabilized with 5 mol% yttria. J. Mech. Behav. Biomed. Mater..

[38] Labetić A., Klaser T., Skoko Ž., Jakovac M., Žic M. (2024). Flexural strength and morphological study of different multilayer zirconia dental materials. Materials.

[39] McLean J.W., von Fraunhofer J.A. (1971). The estimation of cement film thickness by an in vivo technique. Br. Dent. J..

[40] Schwindling F.S., Bechtel K.N., Zenthöfer A., Handermann R., Rammelsberg P., Rues S. (2022). In-vitro fit of experimental full-arch restorations made from monolithic zirconia. J. Prosthodont. Res..

[41] Bihn S.K., Son K., Son Y.T., Dahal R.H., Kim S., Kim J., Hwang J.H., Kwon S.M., Lee J.H., Kim H.D. (2023). In vitro biofilm formation on zirconia implant surfaces treated with femtosecond and nanosecond lasers. J. Funct. Biomater..

[42] Rattanapitak R., Thong-Ngarm W. (2025). Human gingival fibroblast response on zirconia and titanium implant abutment: A systematic review. J. Prosthodont..

[43] Kunii J., Hotta Y., Tamaki Y., Ozawa A., Kobayashi Y., Fujishima A., Miyazaki T., Fujiwara T. (2007). Effect of sintering on the marginal and internal fit of CAD/CAM-fabricated zirconia frameworks. Dent. Mater. J..

